# Cerebellar hemorrhagic injury in premature infants occurs during a vulnerable developmental period and is associated with wider neuropathology

**DOI:** 10.1186/2051-5960-1-69

**Published:** 2013-10-21

**Authors:** Krista M Haines, Wei Wang, Christopher R Pierson

**Affiliations:** 1 Department of Pediatrics, Nationwide Children’s Hospital, Columbus, USA; 2 The Biostatistics Core, Nationwide Children’s Hospital, Columbus, USA; 3 Department of Pathology & Laboratory Medicine, J0359 Nationwide Children’s Hospital, 700 Children’s Drive, Columbus, OH 43205, USA; 4 The Ohio State University College of Medicine, Columbus, USA

**Keywords:** Cerebellar hemorrhagic injury, Cerebellum, Inferior olivary nucleus, Dentate, Neuronal loss, Gliosis

## Abstract

**Background:**

Cerebellar hemorrhagic injury (CHI) is being recognized more frequently in premature infants. However, much of what we know about CHI neuropathology is from autopsy studies that date back to a prior era of neonatal intensive care. To update and expand our knowledge of CHI we reviewed autopsy materials and medical records of all live-born preterm infants (<37 weeks gestation) autopsied at our institution from 1999–2010 who had destructive hemorrhagic injury to cerebellar parenchyma (n = 19) and compared them to matched non-CHI controls (n = 26).

**Results:**

CHI occurred at a mean gestational age of 25 weeks and involved the ventral aspect of the posterior lobe in almost all cases. CHI arose as a large hemorrhage or as multiple smaller hemorrhages in the emerging internal granule cell layer of the developing cortex or in the nearby white matter. Supratentorial germinal matrix hemorrhage occurred in 95% (18/19) of CHI cases compared to 54% (14/26) of control cases (p = 0.003). The cerebellar cortex frequently showed focal neuronal loss and gliosis (both 15/19, 79%) in CHI cases compared to control cases (both 1/26, 4% p < 0.0001). The cerebellar dentate had more neuronal loss (8/15, 53%) and gliosis (9/15, 60%) in CHI cases than controls (both 0/23, 0%; p < 0.0001). The inferior olivary nuclei showed significantly more neuronal loss in CHI (10/17, 59%) than in control cases (5/26, 19%) (p = 0.0077). All other gray matter sites examined showed no significant difference in the incidence of neuronal loss or gliosis between CHI and controls.

**Conclusions:**

We favor the possibility that CHI represents a primary hemorrhage arising due to the effects of impaired autoregulation in a delicate vascular bed. The incidences of neuronal loss and gliosis in the inferior olivary and dentate nuclei, critical cerebellar input and output structures, respectively were higher in CHI compared to control cases and may represent a transsynpatic degenerative process. CHI occurs during a critical developmental period and may render the cerebellum vulnerable to additional deficits if cerebellar growth and neuronal connectivity are not established as expected. Therefore, CHI has the potential to significantly impact neurodevelopmental outcome in survivors.

## Background

Cerebellar injury is a frequent complication of premature birth that is receiving more attention due, in part, to the emergence of improved imaging techniques with enhanced sensitivity to detect posterior fossa lesions [1-3]. Often these injuries have a significant hemorrhagic component and the term cerebellar hemorrhagic injury (CHI) has been applied [3,4]. The actual incidence of CHI is difficult to estimate as it has likely been underdiagnosed clinically [5]. Previous autopsy studies suggest an incidence of 10 to 25%, although this is likely high [6] as recent neuroimaging based studies indicate a much lower incidence [3,4,7-13]. The largest series reported an overall incidence of approximately 3%, which increased to 8.7% in infants with a birth weight less than 750 gm, and decreased in infants of greater birth weight suggesting that the smallest and most premature infants are at greatest risk to develop CHI [4,13].

During this developmental period the human cerebellum is undergoing rapid growth and many complex developmental processes are taking place that are essential to proper cerebellar function [14]. In this period cerebellar growth is rapid and mostly due to the massive increase in external granule cell number. Other critical developmental events occurring in this time period include granule cell migration and the early establishment of cerebellar neuronal circuitry. Because all of these important events occur within this time frame it is considered to represent a critical period of cerebellar development when the cerebellum is vulnerable to injury. An injury at this time could have consequences beyond the direct impact of the damaged cerebellar tissue if the injury impairs or arrests later developmental processes and takes the cerebellum off its developmental trajectory. Because CHI tends to affect the most premature infants during a time when the cerebellum is developmentally vulnerable, CHI could have significant neurodevelopmental impact on survivors, and there is a critical need to learn as much as possible about the neuropathology of CHI.

The number of CHI autopsy studies is limited and most were performed in a previous era of neonatal intensive care and therefore may not be directly applicable to contemporary standards of care and technology [6,15-18]. Autopsy data have suggested that CHI is a primary hemorrhage [18] or possibly due to alterations in venous drainage [17], however, the goal of the latter study was to test the potential impact of a type of ventilation mask attachment that is no longer in use, rather than study CHI itself. Previous autopsy studies indicate that CHI often coexists with a supratentorial germinal matrix hemorrhage (GMH)/ intraventricular hemorrhage (IVH) and suggest that CHI and GMH share common pathogenetic factors [6,15-18]. In one study, 34% of the CHI cases had periventricular leukomalacia (PVL) [17]. Aside from this, the available autopsy studies are mostly silent on whether or not CHI is associated with injury to other neuroanatomic structures such as the pre- and post-cerebellar nuclei, which are of critical importance to cerebellar function. Nonetheless, there are data to suggest that CHI is indeed associated with such injury. Takashima approached the issue from the standpoint of inferior olivary nuclear injury in his study of 15 premature infants who sustained neuronal loss and gliosis to this structure; he observed “a close relationship between lesions of the inferior olivary nuclei and the presence of cerebellar hemispheric lesions such as cerebellar hemorrhage…” [19]. Other autopsy studies indicate that premature infants sustain diverse types of injury to the cerebellar cortex, cerebellar dentate, basis pontis, and inferior olivary nuclei, which is often, but not always, associated with PVL [20]. The impact and extent of CHI on the cerebellum and its associated nuclei are likely of significant clinical importance to survivors and our knowledge of CHI needs to be updated by studying a contemporary CHI patient autopsy dataset.

The cerebellum is known to integrate sensory information and function in motor control, however it also appears to have roles in cognition, learning, behavior and language domains [21-24]. The success of innovations in neonatal critical care means that infants who are most at risk to develop CHI frequently survive and are at increased risk to bear the long-term consequences of CHI, which may contribute to the cognitive, learning and behavioral issues known to affect survivors of premature birth [3,7,10,25]. The high frequency of CHI in premature infants and the vulnerable nature of the cerebellum in this developmental period need to be considered with emerging concepts of cerebellar function as we update our knowledge of CHI neuropathology.

In this study we examined a modern cohort of premature infants with CHI, we sought to determine: 1) the extent and region of the cerebellum involved by CHI; and 2) if CHI is associated with additional neuropathology, especially gray matter lesions that may provide insight into the neurodevelopmental impairments encountered in surviving premature infants. To address these aims we reviewed autopsy materials and medical records of all live-born preterm infants (< 37 weeks gestation) autopsied at our institution from 1999–2010 and identified 19 premature infants who had CHI as defined by the presence of a destructive hemorrhage located in cerebellar parenchyma. The CHI group was compared to a matched control group of 26 premature infants who did not have CHI and were autopsied in the same time frame. We hypothesized that CHI has a characteristic pattern of distribution, and that infants with CHI have a significantly greater incidence of injury to pre- and post-cerebellar nuclei critical to cerebellar processing, i.e. inferior olivary nuclei, basis pontis and cerebellar dentate, compared to control infants who did not sustain CHI.

## Methods

### Case selection

The autopsy records and pathology materials of all premature infants (<37 gestational weeks at birth) autopsied in 1999–2010 at Nationwide Children’s Hospital were retrospectively reviewed. CHI was defined as a destructive lesion of cerebellar parenchyma that was predominately hemorrhagic in nature and appeared to arise from the cerebellum. A group of gender and age-matched subjects who did not have CHI and were autopsied in the same year as a CHI subject were also identified as a control group. Subjects with chromosomal aberrations or other genetic conditions and patients with significant malformations were excluded. To prevent bias all potential control cases meeting these criteria were included. Parental consent for autopsy was granted in each case and the study was performed after approval of the project by the Institutional Review Board at Nationwide Children’s Hospital.

### Medical chart review

The medical chart of each subject was reviewed for key demographic data as well as maternal and neonatal clinical factors. Basic demographic data including maternal age, gestational age, postnatal age, postconceptional age, birth weight, birth length, head circumference, 1 and 5 minute Apgar scores, gender, and race (African-American, Caucasian and Biracial) were recorded. The occurrence of important pregnancy complications such as pregnancy induced hypertension, pre-ecclampsia, ecclampsia, maternal infection around the time of delivery and antibiotic use, prolonged rupture of membranes, preterm labor, chorioamnionitis, abruption, administration of prenatal corticosteroids, and multiple gestation were recorded. Delivery conditions such as the performance of a caesarian section or an emergent delivery were documented. The medical record was also reviewed for neonatal treatment measures such as resuscitation, positive pressure ventilation (PPV), conventional mechanical ventilation (CMV) and intubation, chest compressions and epinephrine administration, high frequency oscillatory ventilation (HFOV), continuous positive airway pressure (CPAP), nasal cannula (NC), intravenous nitrous oxide (iNO), volume expanders (normal saline), vasopressors (dopamine, dobutamine, epinephrine), blood transfusions (packed red blood cells, platelets, plasma) hydrocortisone, sodium bicarbonate, acidosis (< pH 7.20), furosemide and renal failure. The medical record was reviewed for the occurrence of complications of prematurity such as respiratory distress syndrome, bronchopulmonary dysplasia, pneumonia, pneumothorax, pulmonary hemorrhage, sepsis (clinical blood cultures positive for microbial growth and clinical signs), disseminated intravascular coagulation, intestinal injury (small intestinal perforation (SIP), necrotizing enterocolitis (NEC)), surgery (exploratory laparotomy for SIP or NEC) and hyperbilirubinemia. A patent ductus arterious (PDA) was associated with CHI in a previous multivariate analysis, so the identification of PDA on echocardiogram and management measures such as indomethacin prophylaxis and treatment and ibuprofen administration, and the need for surgical intervention to close a PDA was noted from the medical record [4]. The need for pain medication (fentanyl, morphine) or sedatives (Ativan, versed) and the occurrence of seizures were recorded. Body weight, body length, head circumference, and brain mass were recorded from the autopsy reports.

### Review of pathology materials

A mean of 10 hematoxylin-eosin (H&E) stained histopathologic sections were examined from each case. Our institution routinely samples the brain to include neocortex from the frontal lobe (watershed areas) and triple watershed area, the thalamus (at the level of the lateral geniculate nucleus and dorsal medial nucleus), caudate, putamen, globus pallidus, hippocampus (at the level of the lateral geniculate nucleus), cerebellar cortex, cerebellar dentate, midbrain, pons, rostral and caudal medulla and spinal cord.

Autopsy reports and gross photographs were reviewed for the presence of primary hemorrhagic lesions of the cerebellum and to assess the laterality and extent of CHI. Hemorrhages from a supratentorial source could be reliably discerned from a primary cerebellar parenchymal hemorrhage as blood from the former formed a cast of the cerebellomedullary cistern, whereas blood from the later adopted the shape of the cerebellum and was often at least partially covered by leptomeninges. Histopathologic sections were assessed to determine the relative age of the hemorrhage. Acute lesions were defined by the presence of recent hemorrhage with relatively intact red blood cells. Subacute hemorrhages had macrophages that often contained hemosiderin and early organization with tissue fragmentation; chronic lesions were defined as those with cyst-like cavities.

Histopathologic sections were examined for acute neuronal necrosis, neuronal loss and gliosis in the most severely affected high power fields of each gray matter site. Acute neuronal necrosis was defined as neuronal hypereosinophilia with nuclear pyknosis or as karyorrhexis in the case of immature neurons with relatively less cytoplasm. The CHI and control cases all had varying degrees of acute neuronal necrosis in a number of neuroanatomic structures. We considered these findings to represent agonal changes developing in the 24 to 48 hour period prior to death and, instead focused our analysis on neuronal loss and gliosis, which are changes indicative of an injury occurring 3 to 5 days or more prior to death and may more appropriately represent the neuropathology encountered in CHI survivors [26]. Gliosis was defined as the occurrence of 11 or more reactive astrocytes with abundant eosinophilic cytoplasm and an eccentrically situated nucleus with delicate chromatin in a high powered field. Neuronal loss was recognized as focal or confluent areas of neuronal drop out in gray matter structures and was often accompanied by reactive gliosis. We selected these parameters for gliosis and neuronal loss because we felt that they are readily evident using standard histopathological techniques and because they likely represent significant injury as has been shown previously [20]. Gliosis and neuronal loss were evaluated in the entire gray matter structure that appeared in the section and we assessed the mostly severely affected area.

The cerebral white matter of all lobes, corpus callosum, internal capsules and cerebellum was reviewed for evidence of PVL, which was defined as the presence of focal white matter necrosis and diffuse reactive gliosis of the surrounding white matter [27]. The presence of GMH and supratentorial IVH were also analyzed. Pontosubicular necrosis was defined as the presence karyorrhectic neurons in the basis pontis and subiculum of the hippocampus.

### Statistical analysis

The 45 cases were divided into a CHI group (*n* = 19) and a control group (*n* = 26) that did not have CHI. Categorical data were compared between the groups by using likelihood ratio Chi-Square test or Fisher’s Exact test when it was appropriate. Continuous data were compared using either a t test or the nonparametric Wilcoxon two-sample test as appropriate. Significance was defined as a *p* value < 0.05 in all statistical tests. All tests were conducted using SAS 9.3 (SAS Institute Inc., Cary, NC, USA).

## Results

### Clinical findings

Nineteen cases fulfilled the criteria for CHI while 26 cases showed no CHI and were used as controls. There were no significant differences in gender, gestational age at birth, or postconceptional age at death between the CHI and non-CHI groups (Table 1). Birth length in the CHI group (31.91 ± 3.03 cm) was significantly longer compared to controls (34.02 ± 4.07 cm) (p = 0.0376); however, there was no significant difference in birth weight or head circumference (Table 1). The mean 1 minute Apgar score in both groups was approximately 3.5, while the mean 5 minute Apgar score was 5.1 in the CHI group and 5.6 in the control group, which was not a significant difference (Table 1). Somatic and brain measurements at autopsy showed no difference between the two groups (Table 1). The incidence of various maternal factors such as age, pregnancy induced hypertension, preeclampsia, ecclampsia, infection and antibiotic use, prolonged rupture of membranes, chorioamnionitis, multiple gestation, abruption and prenatal corticosteroids did not differ between the two groups (Table 1). The incidence of cesarean section and emergent delivery were higher in the non-CHI group, however, this difference was not significant (Table 1). Of all the clinical factors pertaining to neonatal course examined only pulmonary hemorrhage (CHI: 8/19, 41% vs. control: 3/26, 12%; p = 0.0333), sepsis (CHI: 17/19, 89% vs. control: 15/26, 58%; p = 0.0152) and renal failure (CHI: 14/19, 74% vs. control: 11/26, 42%; p = 0.0339) were significantly different between the two groups (Table 1). Prior multivariate analysis identified PDA as an independent risk factor for CHI in survivors [4], but we noted no significant difference in the incidence of PDA at autopsy. There was no significant difference in the use of various modalities to medically manage a PDA including indomethacin prophylaxis or treatment, or ibuprofen administration or in the incidence of surgical ligation to close a PDA. However, the incidence of pulmonary hemorrhage, a sign of a hemodynamically significant PDA was significantly increased in the CHI group (Table 1) [28].

**Table 1:** Demographic, maternal, and neonatal clinical data

	**CHI**	**Control**	***p *** **value**
**Demographics**			
Gender: Female	(8/19) 42%	(10/26) 38%	0.8053
Gender: Male	(11/19) 58%	(16/26) 62%	
Gestational Age (weeks)	24.98 (2.41)	25.88 (2.29)	0.1318
Postnatal Age (weeks)	2.96 (2.71)	2.88 (3.01)	0.6789
Postconceptional Age (weeks)	27.83 (3.50)	28.81 (3.17)	0.3362
Race: African-American	(6/17) 35%	(9/24) 38%	1.0
Race: Caucasian	(10/17) 59%	(14/24) 58%	
Race: Biracial	(1/17) 6%	(1/24) 4%	
Birth weight (gm)	746.21 (255.16)	808.54 (228.71)	0.1383
Birth length (cm)	31.91 (3.03)	34.02 (4.07)	**0.0376**
Birth head circumference (cm)	22.79 (2.57)	23.26 (2.46)	0.5039
Apgar 1 minute	3.53 (2.29)	3.5 (2.34)	0.8701
Apgar 5 minutes	5.12 (2.23)	5.62 (2.40)	0.4593
Postmortem body weight (gm)	1,203.74 (493.93)	1,209.72 (496.38)	0.9683
Postmortem body length (cm)	34.08 (3.04)	35.0 (4.51)	0.4475
Postmortem head circumference (cm)	24.95 (3.31)	25.20 (2.97)	0.7864
Postmortem brain weight, fixed (gm)	115.47 (36.89)	131.77 (41.54)	0.1909
**Maternal Factors**			
Maternal Age (years)	24.68 (4.41)	26.12 (7.02)	0.407
Pregnancy induced hypertension	(3/19) 16%	(1/26) 4%	0.2954
Pre-ecclampsia/ecclampsia	(1/19) 5%	(2/24) 8%	1.0
Maternal infection/antibiotics	(9/19) 47%	(12/26) 46%	0.9357
Preterm labor	(16/19) 84%	(26/26) 100%	0.909
Prolonged rupture of membranes	(5/19) 26%	(7/26) 27%	0.9637
Chorioamnionitis	(6/19) 32%	(8/26) 31%	0.9538
Multiple gestation	(4/19) 21%	(7/26) 27%	0.7363
Abruption	(7/19) 37%	(5/26) 19%	0.1870
Prenatal steroids	(15/19) 79%	(18/26) 69%	0.4666
**Neonatal Course**			
Cesarean section	(8/19) 42%	(18/26) 69%	0.0688
Emergent delivery	(11/19) 58%	(18/26) 69%	0.4327
Surfactant	(19/19) 100%	(25/26) 96%	1.0
iNO	(6/19) 32%	(7/26) 27%	0.7341
Chest compressions and/or epinephrine	(5/19) 26%	(3/26) 12%	0.2528
Mechanical ventilation	(19/19) 100%	(26/26) 100%	1.0
Positive pressure ventilation (PPV) and intubation	(19/19) 100%	(25/26) 96%	1.0
High frequency oscillatory ventilation (HFOV)	(14/19) 74%	(17/26) 65%	0.5525
Continuous positive airway pressure (CPAP)/nasal cannula	(9/19) 47%	(14/26) 54%	0.6677
Hypoxia	(19/19) 100%	(3/26) 12%	0.2515
Pneumonia	(6/19) 32%	(5/26) 19%	0.4851
Respiratory distress syndrome (RDS)	(19/19) 100%	(25/26) 96%	1.0
Bronchopulmonary dysplasia (BPD)	(5/19) 26%	(8/26) 31%	0.7448
Pneumothorax	(2/19) 11%	(7/26) 27%	0.2644
Pulmonary hemorrhage	(8/19) 41%	(3/26) 12%	**0.0333**
PDA on ultrasound	(11/17) 65%	(11/21) 53%	0.4442
Indomethacin prophylaxis	(14/19) 74%	(17/26) 65%	0.5525
Ibuprofen/indomethacin	(0/19) 0%	(4/26) 15%	0.1264
Surgical ligation of PDA	(3/19) 16%	(1/26) 4%	0.2954
Vasopressors	(19/19) 100%	(23/26) 88%	0.2515
Hydrocortisone	(13/19) 68%	(13/26) 50%	0.2166
Volume expanders	(19/19) 100%	(24/26) 92%	0.5010
Blood transfusion	(19/19) 100%	(25/26) 96%	1.0
Hyperbilirubinemia	(18/18) 100%	(23/24) 96%	1.0
Disseminated intravascular coagulation	(11/19) 58%	(10/26) 38%	0.1968
Sepsis	(17/19) 89%	(15/26) 58%	**0.0152**
Meningitis	(0/19) 0%	(0/26) 0%	1.0
Intestinal injury	(10/19) 53%	(12/26) 46%	0.6677
Surgery	(9/19) 47%	(8/26) 31%	0.3532
Feeds	(12/19) 63%	(15/26) 58%	0.7660
Total parental nutrition	(19/19) 100%	(26/26) 100%	1.0
Bicarbonate administered	(19/19) 100%	(25/26) 96%	1.0
Acidosis (pH < 7.20)	(19/19) 100%	(26/26) 100%	1.0
Renal failure	(14/19) 74%	(11/26) 42%	**0.0339**
Furosamide	(9/19) 47%	(11/26) 42%	0.7358
Pain/sedation medications	(19/19) 100%	(22/26) 85%	0.1264
Seizures	(5/19) 26%	(7/26) 27%	0.9637

Data reported as percentages for categorical variables. For continuous variables data are reported as mean and (standard deviation). *p* values in bold denote statistical significance.

### Cerebellar hemorrhage

**Figure 1:**
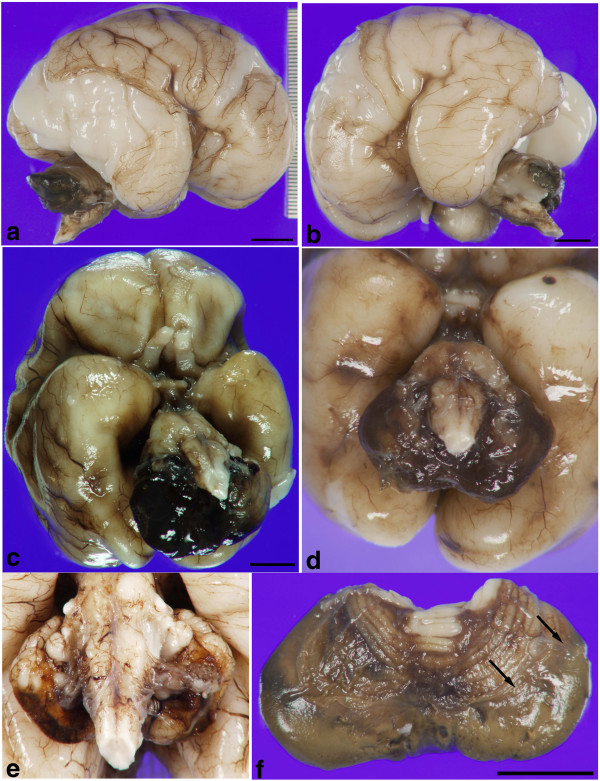
**Gross photographs demonstrating that CHI involves the ventral surface of the cerebellum. a** Right and **b** left lateral views of CHI involving the inferior aspect of the cerebellum in an infant who was 25 weeks gestational age and survived 3 weeks. The left cerebellar hemisphere is partially removed in **b** to show the depth of involvement by the hematoma. **c** View of CHI from basal surface of the brain depicted in panels **a** and **b** and **d** an infant who was 25 weeks gestation and survived 3 weeks demonstrate involvement of the ventral surface of the cerebellum. **e** Close up view of the ventral aspect of the cerebellum from a chronic case of CHI with brown-tinged leptomeninges due to hemosiderin in an infant born at 24.5 weeks gestation and survived 10 weeks. **f** Superior view of the cerebellum detached from the brainstem showing relative sparing of the superior folia in an infant born at 27.5 weeks gestation and survived 5 weeks. Bar is 1 cm in all panels.

**Figure 2:**
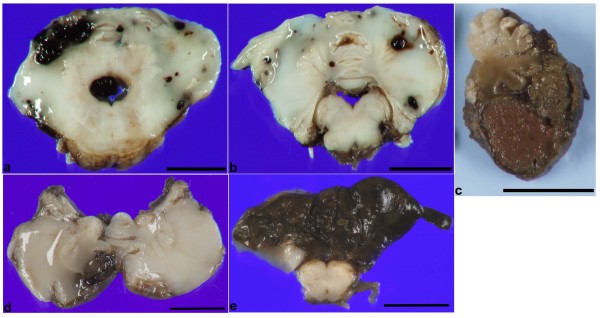
**Gross photographs of the cut surface of CHI. a** Horizontal sections at the level of the mid pons and **b** rostral medulla showing multifocal hemorrhages and a crescentic larger superficial hemorrhage in panel **a** from a 29 weeks gestation infant who survived 3 weeks. **c** Sagittal section through the cerebellar hemorrhage in Figure 1f showing replacement of inferior cerebellar tissue by a hematoma that is partially covered by leptomeninges. The remaining cerebellar cortex is atrophic. **d** Unilateral CHI in an infant born at 30.5 weeks gestation who survived 1 week. Note the dusky appearance of the hemisphere involved by the hemorrhage relative to the uninvolved contralateral hemisphere. **e** Horizontal section at the level of the rostral medulla showing near total replacement of the cerebellum by hematoma covered with leptomeninges. Bar is 1 cm in all panels.

In all cases a destructive hematoma occupied the inferior aspect of the posterior lobe of the cerebellum (Figure 1a-f), in a distribution that roughly corresponded to the territory supplied by the posterior inferior cerebellar artery (PICA). The hematomas involved the superficial cortex and white matter (Figure 1b) and at times were at least partially covered by leptomeninges (Figure 1c,d). Grossly most cases were characterized by fresh hemorrhage (Figure 1a-d), however, one case showed chronic changes with yellow-tinged leptomeninges and mild cerebellar atrophy (Figure 1e). While the inferior surface of the posterior lobe was always involved many cases showed varying degrees of involvement superiorly (Figure 1b,f). The cut surface frequently showed multifocal hemorrhages in the white matter subjacent to the cortex that were separate from the main hematoma (Figure 2a,b). In some cases, significant portions of the cerebellar parenchyma were replaced by hematoma and covered with leptomeninges (Figure 2c-e). Fourteen cases had bilateral involvement of the hemispheres and vermis, while 5 cases showed unilateral involvement of a cerebellar hemisphere (Figure 2d).

**Figure 3:**
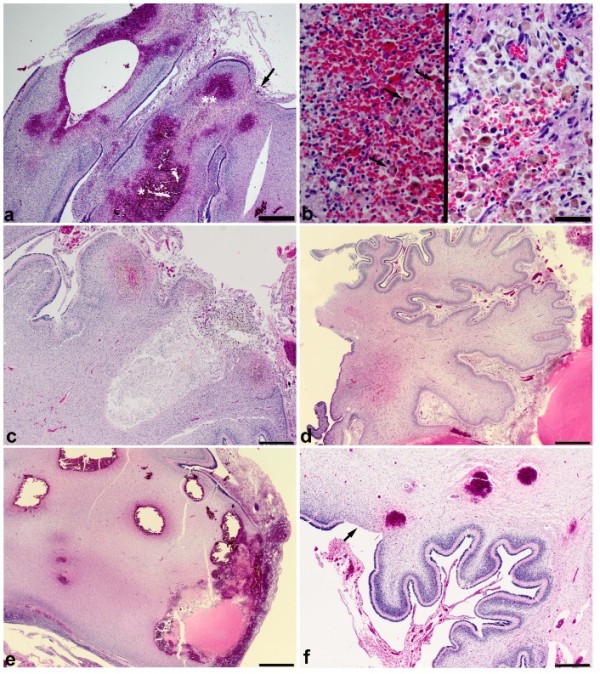
**Histopathology of CHI demonstrates a multifocal hemorrhagic process and is associated with cortical hypoxic-ischemic injury or atrophy. a** Multiple hemorrhages arising from different folia with multifocal extension into the leptomeninges in an infant of 25 weeks gestation who survived about 3 weeks. There are multiple areas of focal cortical loss, one of which is indicated by an arrow. Hemorrhages appear to be centered in the folia and show a mixture of acute and subacute changes suggesting that CHI may be due to repeated hemorrhagic episodes. **b** Left panel shows a higher magnification of the area marked by * in **a** demonstrating acute hemorrhage with scattered hemosiderin-laden macrophages (arrows). Right panel shows the area marked by ** in **a** at higher magnification demonstrating many hemosiderin-laden macrophages in an organizing cavity. **c** Chronic CHI with cavitation, hemosiderin and cholesterol clefts in the brain depicted in Figure 1e. **d** Atrophic cerebellar cortex uninvolved by hemorrhage from the sagittal section depicted in Figure 2c. **e** A larger hemorrhage extending into the leptomeninges with other smaller hemorrhages nearby in the white matter suggesting that the larger hemorrhages may represent a coalescence of multiple smaller hemorrhages (gross pathology is depicted in Figure 2a,b). **f** Three acute hemorrhages in the deep cortex and superficial white matter in a 29 weeks gestation infant who survived 3 weeks. The focal loss of gliotic cerebellar cortex (arrow) is consistent with a hypoxic-ischemic episode. All photomicrographs are taken from hematoxylin and eosin stained sections. **a** bar is 500 μm. **b** bar is 50 μm. **c** bar is 1 mm. **d- f** bar is 500 μm.

Histopathologically, 8/19 cases demonstrated only acute hemorrhage while 11/19 cases showed acute hemorrhage admixed with subacute or chronic changes. Ten cases showed subacute changes such as tissue fragmentation associated with infiltration by foamy and hemosiderin-laden macrophages admixed with acute hemorrhages with intact red blood cells (Figure 3a,b). One CHI case had chronic changes consisting of cavitated spaces containing hemosiderin-laden macrophages and cholesterol clefts (Figure 3d) accompanied by other areas with acute and/or subacute hemorrhage. Often the chronic cases showed atrophy of folia with loss of the external granule layer in areas not overtly involved by hemorrhage (Figure 3d). The hemorrhages typically appeared in the white matter or in the cerebellar cortex near the junction of white matter and the emerging internal granule layer (Figure 3a,e,f). Often these hemorrhages were multifocal with satellite hemorrhages appearing near a larger hemorrhage (Figure 3e) or with multiple hemorrhages of comparable size clustered together (Figure 3a,f). The cerebellar cortex in the CHI cases showed significantly more neuronal loss (15/19, 79%) and gliosis (15/19, 79%) than controls (1/26, 4% for both neuronal loss and gliosis) (both p < 0.0001) (Table 2) (Figure 3a,c,d-f). The germinal matrix of the fourth ventricle was sampled in 3 of the CHI cases and one case showed a focal acute unilateral hemorrhage confined to the matrix. The external granule layer showed a focal acute hemorrhage in 2/19 CHI cases, which were always accompanied by larger hemorrhages that were more deeply located in the cerebellum.

**Table 2:** Neuropathologic findings

**Neuropathological finding**	**CHI**	**Control**	***p *** **value**
Periventricular leukomalacia	(5/19) 26%	(1/26) 4%	0.0687
Germinal matrix hemorrhage	(18/19) 95%	(14/26) 54%	**0.003**
Intraventricular hemorrhage Grades 3 or 4	(12/18) 67%	(10/14) 71%	1.0
Pontosubicular necrosis	(11/16) 69%	(7/26) 27%	**0.0073**
Frontal cortex - neuronal loss	(0/19) 0%	(0/26) 0%	NA
Frontal cortex - gliosis	(0/19) 0%	(0/26) 0%	NA
Parietal cortex - neuronal loss	(0/17) 0%	(0/24) 0%	NA
Parietal cortex - gliosis	(0/17) 0%	(0/24) 0%	NA
Temporal cortex - neuronal loss	(0/19) 0%	(1/24) 4%	1.0
Temporal cortex - gliosis	(0/19) 0%	(1/23) 4%	1.0
Occipital cortex - neuronal loss	(0/5) 0%	(0/2) 0%	NA
Occipital cortex - gliosis	(0/5) 0%	(0/2) 0%	NA
Putamen – neuronal loss	(1/18) 6%	(2/24) 8%	1.0
Putamen – gliosis	(1/18) 6%	(2/24) 8%	1.0
Caudate – neuronal loss	(1/17) 5%	(3/26) 12%	1.0
Caudate – gliosis	(1/17) 5%	(3/26) 12%	1.0
Globus pallidus – neuronal loss	(0/14) 0%	(0/13) 0%	NA
Globus pallidus – gliosis	(0/14) 0%	(2/13) 15%	0.2222
Thalamus – neuronal loss	(2/16) 13%	(5/22) 23%	0.6754
Thalamus – gliosis	(2/16) 13%	(5/22) 23%	0.6754
Hypothalamus- neuronal loss	(0/5) 0%	(2/3) 66%	0.1071
Hypothalamus – gliosis	(0/5) 0%	(2/3) 66%	0.1071
Hippocampus – neuronal loss	(0/19) 0%	(1/22) 5%	1.0
Hippocampus – gliosis	(1/19) 5%	(1/22) 5%	1.0
Midbrain, tectum – neuronal loss	(6/17) 35%	(5/24) 21%	0.4757
Midbrain, tectum – gliosis	(6/17) 35%	(6/24) 25%	0.5072
Midbrain, tegmentum – neuronal loss	(0/17) 0%	(1/25) 4%	1.0
Midbrain, tegmentum – gliosis	(0/17) 0%	(2/25) 8%	0.5064
Pons, tegmentum – neuronal loss	(2/19) 11%	(1/24) 4%	0.5751
Pons, tegmentum – gliosis	(5/19) 26%	(1/24) 4%	0.0723
Rostral medulla, tegmentum – neuronal loss	(3/18) 17%	(2/26) 8%	0.3859
Rostral medulla, tegmentum – gliosis	(4/19) 21%	(7/26) 27%	0.7363
Inferior olivary nucleus – neuronal loss	(10/17) 59%	(5/26) 19%	**0.0077**
Inferior olivary nucleus - gliosis	(17/18) 94%	(18/26) 69%	0.0603
Cerebellar cortex – neuronal loss	(15/19) 79%	(1/26) 4%	**<0.0001**
Cerebellar cortex – gliosis	(15/19) 79%	(1/26) 4%	**<0.0001**
Dentate nucleus – neuronal loss	(8/15) 53%	(0/23) 0%	**<0.0001**
Dentate nucleus - gliosis	(9/15) 60%	(0/23) 0%	**<0.0001**
Spinal cord – neuronal loss	(0/16) 0%	(1/23) 4%	1.0
Spinal cord - gliosis	(0/16) 0%	(1/23) 4%	1.0

Data reported as percentages for categorical variables. NA, statistical testing not applied. Hippocampus refers to all parts of Ammon’s horn, but excludes subiculum. *p* values in bold denote statistical significance.

### Associated neuropathologic findings

The CHI cases had a higher incidence of PVL (5/19, 26%) than controls (1/26, 4%) but the difference was not significant (p = 0.0687) (Table 2). At autopsy, 95% (18/19) of CHI cases had a GMH compared to 54% (14/26) of controls, which was a significant difference (p = 0.003) (Table 2) [29]. Grade 3 and 4 intraventricular hemorrhages occurred in 12 of the 18 CHI cases (67%) and in 10 of the 14 control cases that had a GMH (71%) (p = 1.0) (Table 2). Pontosubicular necrosis is a common pattern of injury in this age group and it occurred in 69% (11/16) of CHI cases compared to 27% (7/26) of controls, a difference that was significant (p = 0.0073) (Table 2).

**Figure 4:**
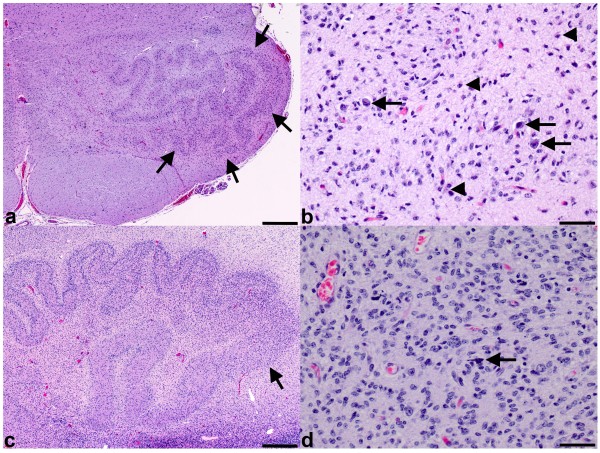
**CHI is associated with significant pathology in the inferior olivary and cerebellar dentate nuclei. a** Low powered magnification shows increased cellularity of the anteriolateral inferior olivary nucleus (arrows) from the brain depicted in Figure 2e. The increased cellularity makes the edges of the anteriolateral olivary ribbon appear ill-defined relative to the medial aspect of the olivary ribbon. **b** High powered magnification of the anteriolateral inferior olivary nucleus illustrates that this increased cellularity is due to numerous reactive astrocytes (arrowheads) accompanied by significant neuronal loss with only a few scattered neurons remaining (arrows). **c** Low powered magnification of the cerebellar dentate nucleus demonstrating an area (arrows) where the border between the nucleus and white matter is obscured due to increased cellularity (arrows). **d** High powered magnification of the dentate nucleus shows that this increase in cellularity is due to an abundance of reactive astrocytes. There are scattered mineralizations (arrows), but no surviving neurons. All photomicrographs are taken from hematoxylin and eosin stained sections. **a** and **c** bar is 500 μm. **c** and **d** bar is 50 μm.

The CHI and control groups showed no significant difference in the incidence of neuronal loss or gliosis in the frontal, parietal, temporal, and occipital cortices, putamen, caudate, globus pallidus, thalamus, hypothalamus, hippocampus (except subiculum) and spinal cord (Table 2). The tectum and tegmentum of the midbrain, pons and rostral medulla of the CHI and control groups also showed no significant difference in the incidence of neuronal loss or gliosis (Table 2). The inferior olivary nucleus demonstrated a significantly higher incidence of neuronal loss in the CHI cases (10/17, 59%) compared to controls (5/26, 19%) (p = 0.0077) (Figure 4a,b) (Table 2). The incidence of gliosis in the inferior olivary nucleus was also higher in CHI cases (17/18, 94%) compared to controls (18/26, 69%), however, this difference was not statistically significant (p = 0.0603) (Table 2). The cerebellar dentate nucleus showed significantly higher incidences of neuronal loss (8/15, 53%) and gliosis (9/15, 60%) in the CHI group compared to the control group (0/23, 0%, for both neuronal loss and gliosis) (both p < 0.0001) (Figure 4 c,d) (Table 2).

## Discussion

This case-controlled neuropathologic autopsy study of CHI in premature infants shows that CHI tends to be bilateral and involve the ventral surface of the posterior lobe of the cerebellum. CHI is often multifocal and appears to arise in the deep cerebellar cortex or adjacent white matter. We found that CHI occurs in association with significant pathology in the inferior olivary and dentate nuclei, which are critical input and output structures, respectively, of the cerebellar cortex. CHI occurs during a critical period of cerebellar development when the cerebellum may be particularly vulnerable to injury and may account for a component of the adverse neurodevelopmental outcomes of premature birth.

At autopsy, CHI frequently involved the ventral surface of one or both hemispheres and vermis, which is consistent with the pattern observed in severely affected survivors [30,31]; however, unilateral involvement tends to be more common in survivors [4,8], and was only observed in five of our autopsy cases. This may simply reflect the fact that subjects who come to autopsy were generally more severely affected than survivors. The frequency of PVL in this series was 26%, which is similar to that reported in a prior autopsy study (34%), although each study used a slightly different definition of PVL [17]. Nonetheless, the frequency of PVL in CHI subjects at autopsy is likely higher than that reported in survivors [4,17]. A novel finding in this study is the significantly higher incidence of pontosubicular necrosis (69%) in CHI patients compared to controls (27%). Pontosubicular necrosis is rarely noted in isolation, but rather tends to coexist with other hypoxic-ischemic lesions [26]. It is possible that our patient population may not be completely representative of the brain pathology of premature infants with CHI who survive beyond the perinatal period, but nonetheless it still provides important information about CHI itself and may help guide future research into the neuropathologic substrate of the neurological sequelae encountered in CHI survivors.

Nearly all of the infants with CHI also had supratentorial hemorrhage, an association which has been recognized previously [4,6,8,18]. CHI and GMH/IVH commonly coexist, which suggests that these hemorrhagic lesions share common pathogenetic mechanisms. In our series CHI tended to be multifocal and frequently consisted of multiple variably sized hemorrhages of different histopathologic age. This multifocality and heterogeneous histopathologic picture suggests that CHI may develop as a series of recurrent hemorrhagic episodes occurring over a period of time. It is possible that the large main hemorrhage grossly evident in many cases of CHI actually originated from a number of smaller bleeds that coalesced together. If this is indeed the case then CHI may represent an evolving process, rather than a single event. More work is needed on the temporal pace of CHI, but this could be important to take into consideration when instituting future therapeutic or preventative measures.

We were unable to reproduce many of the recently identified risk factors associated with CHI, likely because these studies, unlike ours, were conducted in surviving infants. However, our findings are in accordance with previously noted themes that CHI patients are critically ill and require intense supportive therapy [4,11]. A multivariate analysis indicated that emergency caesarean section, PDA and acidosis were independent risk factors for CHI [4]. At autopsy we observed no statistically significant difference in the frequency of acidosis, caesarean section or emergent delivery between CHI and control infants. Sepsis and renal failure were significantly more frequent in the CHI group compared to controls. The presence of sepsis may indicate a role for infection and the inflammatory cascade, while renal failure could further substantiate the role of poor perfusion in severe CHI cases. At autopsy we noted no difference in the incidence of PDA between the CHI and control cases, or in the frequency of measures taken to close a PDA. Pulmonary hemorrhage, however, is indicative of a hemodynamically significant PDA [28], and it was significantly more frequent in CHI patients than in controls at autopsy and has been reported to be increased in CHI survivors [4]. Middle cerebral artery blood flow velocity is reduced in association with a PDA, so it seems possible that the presence of a PDA could also impair cerebellar perfusion [32]. While the direct effect of a PDA on the posterior cerebral circulation has not been formally evaluated, PDA has been associated with cerebellar infarcts [33]. Overall, hemodynamic factors such as impaired autoregulation and a significant PDA seem to be important in CHI pathogenesis, as they are in GMH and IVH [34,35].

CHI could arise from a number of intra- or extracerebellar sources and likely has a complex, multifactorial pathogenesis with hematologic and vascular factors potentially playing important roles. Donet et al. suggested that cerebellar hemorrhage in premature infants is due to the dissection of blood from either the fourth ventricle or from the subarachnoid space that originates from an IVH [36]. We found no parenchymal tract that would be consistent with this mechanism, but we also made an effort to include only primary cerebellar hemorrhages. It has been suggested that primary cerebellar hemorrhages arise from either the subependymal germinal matrix of the fourth ventricle or from the external granule layer which has a superficial venous anastomoses [6,16,17]. We found little evidence to support either of these sites as a substantial source of hemorrhage. None of our cases showed significant hemorrhage in these locations and those that did were always accompanied by other larger, more deeply situated hemorrhages. The hemorrhages we observed occurred in the deep cortex and in the white matter near the internal granule layer. The internal granule layer is one of, if not the, most highly vascularized region of the human brain [37]; however, the dense vascularity of this region would only enhance the probability of hemorrhage, and other factors must contribute. We suspect that the vessels in the region of the white matter-internal granule layer interface are immature and relatively weak in this developmental period due to the rapid angiogenesis that may be occurring in order to accommodate the quickly expanding internal granule cell population. We are currently testing this hypothesis.

Although it is plausible that CHI represents hemorrhagic conversion of a thromboembolic infarct CHI was often bilateral and infarcts were not noted in other brain vascular distributions, making hemorrhagic conversion of an infarct an unlikely cause of CHI. Autoregulation of cerebral perfusion is impaired in critically ill premature infants and limits their ability to maintain uniform cerebral blood flow across a range of perfusion pressures. Impaired autoregulation renders the brain susceptible to episodes of hypoperfusion/ischemia, which can weaken blood vessels making them prone to rupture during episodes of hyperperfusion leading to hemorrhage. Interestingly, nearly all of the hemorrhages we studied involved the ventral posterior lobe of the cerebellum, which is largely supplied by the PICA. The autoregulatory capacity of the cerebellum is even narrower than that of the cerebrum [38]. Whether the PICA is more susceptible to the hemodynamic perturbations typical of prematurity is unknown; however, the cerebella of CHI cases often demonstrated focal cerebellar cortical loss and gliosis, which is consistent with hypoperfusion (Figure 3a and d).

The ventral cerebellum not only has a distinct arterial supply, it also has a distinct venous return. The superior cerebellar veins drain superior hemispheric and vermal areas to the great cerebral vein of Galen or to the proximal straight sinus. The posterior inferior veins drain the inferior hemispheres into the transverse sinus and the inferior vermis drains into the confluens [39]. The premature infant has a compliant skull and external forces causing occipital compression can displace the squamous portion of the occipital bone under the parietal bones distorting the venous sinuses at the confluens thereby increasing venous pressure, which would preferentially affect the ventral cerebellum [5,39]. This raises the possibility that CHI could arise from a venous source, as some have suggested [17]. The venous drainage of the deep zone of the cerebellar cortex principally occurs via vessels Duvernoy et al. term V5 veins, which have extensive ramifications that drain the internal granule layer and subcortical white matter [37]. The location of many of the hemorrhages in this series corresponds to the location of the V5 veins. It is possible that some cases of cerebellar hemorrhage are due to a venous source; however, the frequent coexistence of CHI with cortical injury that is likely hypoxic-ischemic in nature speaks against a venous source underlying all cases. More research is needed to elucidate the vascular source of CHI.

The cerebellum undergoes a lengthy developmental process that extends well into the postnatal period so cerebellar injury in premature infants can potentially alter the developmental trajectory of the cerebellum. Quantitative MRI studies have indeed demonstrated that premature birth is associated with impaired cerebellar growth [40]. In this series CHI occurred at a mean gestational age of approximately 25 weeks, at which time the cerebellum has achieved only 20 to 25% of its term volume [41]. From 24 gestational weeks to term birth cerebellar growth peaks mostly due to proliferation, migration and differentiation of external granule precursor cells [14]. The death of external granule cells (or their limited proliferation) would reduce the number of internal granule cells and decrease excitatory input to Purkinje cells resulting in defects in cerebellar circuitry. CHI can directly destroy a significant amount of cerebellar tissue and impair cerebellar growth and the establishment of proper neuronal connectivity patterns. However, CHI could also exert indirect effects that are capable of impacting neurodevelopment and outcome. For example, CHI exposes external granule precursor cells on the surface of the molecular layer to non-heme iron and hemosiderin, which can produce free radicals, reactive oxygen species and pro-inflammatory cytokines [42-46]. Subarachnoid blood in premature infants can decrease glutamate transporter expression in Purkinje cells and Bergman glia, leading to excitotoxic cell death via increased extracellular glutamate [47]. Subarachnoid blood can induce vasoconstriction of pial vessels at the surface of the cerebellum in experimental animals, which would further potentiate ischemic injury [46]. Experimental models indicate that meningeal cells over the cerebellar surface are important for proper foliation, neurogenesis and cortical lamination [48-50], therefore hemorrhages disrupting the leptomeninges could impact cerebellar tissue that is not directly involved by CHI. Free radicals, reactive oxygen species, cytokines, excitotoxicity and altered meningeal-cortical interactions are potentially important pathogenetic factors that warrant further study.

We observed significant neuronal loss and gliosis in the dentate and inferior olivary nuclei in association with CHI at autopsy, but not in other gray matter sites (Table 2). This dentate and inferior olivary nuclear pathology likely represents a transsynaptic degenerative phenomenon; as tissue adjacent to these nuclear structures was intact and their overall architecture was recognizable. This is in accordance with a study by Takashima who examined 15 cases with inferior olivary nuclear pathology and observed an association with lesions in the cerebellar hemisphere and further suggested that the olivary pathology is due to transsynaptic degeneration [19]. The dentate and inferior olivary nuclei, like the cerebellar cortex, are in a critical developmental period at 25 weeks gestation [51-54]. Dentate neurons increase in size and dendrites start to branch extensively and acquire spines from 20 to 25 weeks [53]. Extensive infolding of the early dentate nucleus begins at 24 weeks and continues until the mature serpentine configuration is attained at 35 weeks gestation [53]. A slower phase of maturation extends into the neonatal period, during which time the neurons acquire an even more complex dendritic tree [53]. The olivary nuclei are the main origin of climbing fibers, which extend into the cerebellum at about 20 weeks gestation and synapse with Purkinje cells around 28 weeks. At 34 weeks the climbing fibers ascend the Purkinje cell dendrites and this process continues into postnatal life [51,52]. The long developmental period of the cerebellar cortex and its associated nuclei may enable the interconnections between these structures to become more complex or larger in number, and this could increase cerebellar processing capacity [52,53,55]. Since these intricate interconnections are established over an extended period of the time, the cerebellum and its associated nuclei are not only impacted by the immediate destructive effects of CHI, but may also be vulnerable to any potential effects CHI may have on later developmental events, which would be expected to delay or prevent the establishment of critical neuroanatomic connections and contribute to long term neurodevelopmental deficits in survivors. More research is needed to determine specifically how CHI can alter the developmental trajectory of the cerebellum and its associated nuclei.

Survivors of premature birth are at risk for deficits in motor function as well as in cognitive, language, socialization and behavioral domains [21,31,56,57]. The long-term effects of CHI are not fully understood, but there is mounting evidence to support the role of cerebellar injury in the pathogenesis of the non-motor deficits in survivors [3,8,9,21,25,58]. The largest study retrospectively compared CHI patients to age-matched controls and uncovered a significant incidence of non-motor deficits, including disorders in language and cognition as well as social and behavioral issues independent of those associated with supratentorial cerebral injury [25]. Studies report that bilateral CHI as we encountered at autopsy is associated with more severe adverse neurodevelopmental outcomes [10,25]. In terms of topography, hemispheric injury is associated with more severe neurological abnormalities while global pervasive developmental deficits were far more common in patients with injuries to the vermis [25]. The mechanism underlying these associations is unknown and although they may be due to a primary cerebellar defect, cerebellar lesions can exert a negative impact on cerebral development. Limperopoulos et al. demonstrated a relatively symmetric decrease in cerebral volume following bilateral cerebellar hemorrhage, while unilateral cerebellar hemorrhage resulted in a significant reduction in contralateral cerebral volume [59]. Although it seems likely that CHI plays an important role in the neurologic morbidity of premature neonates these disabilities can evolve over time so long-term longitudinal follow-up studies are essential to determine if the deficits associated with CHI endure.

## Conclusions

In conclusion, this study establishes the neuropathology of CHI in an autopsy case controlled cohort of premature infants. Our results indicate that CHI tends to involve the ventral cerebellum and may arise in the deep cerebellar cortex or adjacent white matter. We favor the possibility that CHI arises as a primary bleed due to the impact of impaired autoregulation in a delicate vascular bed. The high frequency of cortical neuronal loss and gliosis further suggests the presence of cerebellar hypoxia-ischemia and is in keeping with this possible mechanism. CHI occurs during a key period of development when the cerebellum and its associated pre- and post-cerebellar cortical nuclei are vulnerable to injury. CHI likely has long-term consequences on future neurodevelopment may account for a significant component of the neurological deficits encountered in survivors of premature birth. These deficits are due not only to the direct damage to the cerebellum, but also to potential indirect effects such as injury to cerebellar rely nuclei and possibly, to the cerebrum, via impaired trophic effects.

## Competing interests

The authors declare that they have no competing interests.

## Authors’ contributions

KMH helped plan the study, collected and interpreted the clinical data, and helped draft portions of the manuscript. WW performed the statistical analysis. CRP planned the study, interpreted the clinical and neuropathological data, and wrote the manuscript. All authors read and approved the manuscript.
